# TAp73 promotes anti-senescence-anabolism not proliferation

**DOI:** 10.18632/aging.100701

**Published:** 2014-11-13

**Authors:** Massimiliano Agostini, Maria Victoria Niklison-Chirou, Maria Valeria Catani, Richard A. Knight, Gerry Melino, Alessandro Rufini

**Affiliations:** ^1^ Medical Research Council, Toxicology Unit, Leicester LE1 9HN, UK; ^2^ Department of Experimental Medicine and Surgery, University of Rome “Tor Vergata”, 00133 Rome, Italy; ^3^ Biochemistry Laboratory IDI-IRCC, c/o Department of Experimental Medicine and Surgery, University of Rome “Tor Vergata”, 00133 Rome, Italy; ^4^ Department of Cancer Studies and Molecular Medicine, University of Leicester, Leicester UK; ^5^ Blizard Institute of Cell and Molecular Science, Barts and the London School of Medicine and Dentistry, Queen Mary University of London, London, E1 2AT, UK; current address

**Keywords:** p73, p53 family, senescence, aging, metabolism, apotosis, cancer

## Abstract

TAp73, a member of the p53 family, has been traditionally considered a tumor suppressor gene, but a recent report has claimed that it can promote cellular proliferation. This assumption is based on biochemical evidence of activation of anabolic metabolism, with enhanced pentose phosphate shunt (PPP) and nucleotide biosynthesis. Here, while we confirm that TAp73 expression enhances anabolism, we also substantiate its role in inhibiting proliferation and promoting cell death. Hence, we would like to propose an alternative interpretation of the accumulating data linking p73 to cellular metabolism: we suggest that TAp73 promotes anabolism to counteract cellular senescence rather than to support proliferation.

## INTRODUCTION

Metabolic adaptation has emerged as a hallmark of cancer and a promising therapeutic target [1-9]. Rapidly proliferating cancer cells adapt their metabolism by increasing nutrient uptake and reorganizing metabolic fluxes to sustain biosynthesis of macromolecules necessary to achieve cell division and maintained redox and energy equilibrium [10-18]. It is increasingly evident that oncogenes and tumor suppressor genes regulate the metabolic rearrangement in cancer cells [19-23].

TAp73 acts as a tumor suppressor [24-27], at least partially through induction of cell cycle arrest and apoptosis [28] and through regulation of genomic stability [29, 30]. In addition, premature senescence is observed in TAp73 null mice suggesting that the presence of TAp73 is necessary to counteract senes-cence [31]. At least in part, this anti-senescence effect is mediated by a direct transcriptional effect of TAp73 on mitochondrial gene Cox4i1, hence regulating mitochondrial metabolism [31]. We also reported that TAp73 induces serine biosynthesis and glutaminolysis in lung cancer cells, via a direct transactivation of GLS2[32]. Interestingly, increased serine biosynthesis sustains cancer growth and has been recently reported to be nourished in breast cancer and melanoma by amplification of phosphoglycerate dehydrogenase gene [33, 34]. Recently, Du and colleagues [35, 36] reported that TAp73 triggers the expression of glucose-6-phosphate dehydrogenase (G6PD), the rate-limiting enzyme of the pentose phosphate pathway (PPP), thus increasing flux through the PPP. By doing so, TAp73 diverts glucose to the production of NADPH and ribose, promoting synthesis of nucleotides and contributing to scavenging of reactive oxygen species [37]. The authors also describes that depletion of TAp73 leads to defective cellular proliferation, promptly rescued by G6PD expression or, alternatively, by addiction of nucleosides and ROS scavengers. Therefore, the authors conclude that TAp73 regulate metabolism with the ultimate result of promoting cell growth and proliferation, in striking contrast to its established role as tumor suppressor.

Prompted by these findings, we attempted to elucidate the regulation of cellular metabolism and proliferation by TAp73 using high throughput metabolomics study upon ectopic expression of TAp73βisoform in human p53-null osteosarcoma cell lines (SaOs-2). Moreover, we validated in-vitro findings, in brain tissue from TAp73 null mice. Here, we report that TAp73 promotes anabolic metabolism and nucleotide biosynthesis. Moreover, our data suggest that TAp73 promotes glycolysis and enhances the Warburg effect. Nonetheless, these changes are unlikely to lead to cell proliferation, as accompanied by robust upregulation of the cell cycle inhibitor p21 and marked apoptosis. Therefore, based on these and other findings we propose that TAp73-mediated control of cellular metabolism should be interpreted on the light of its multifaceted physiological activities, especially in the context of regulation of animal aging, fertility and neurodegerative diseases[31, 38-41]. We suggest that TAp73 promotes a metabolic reprogramming that act to protect from accelerated senescence and aging, as previously demonstrated[31, 42]. This interpretation will reconcile the findings of Du and colleagues with the abundant literature attributing a tumour suppressive function to TAp73.

## RESULTS

### TAp73 activates anabolic pathways

**Figure 1:**
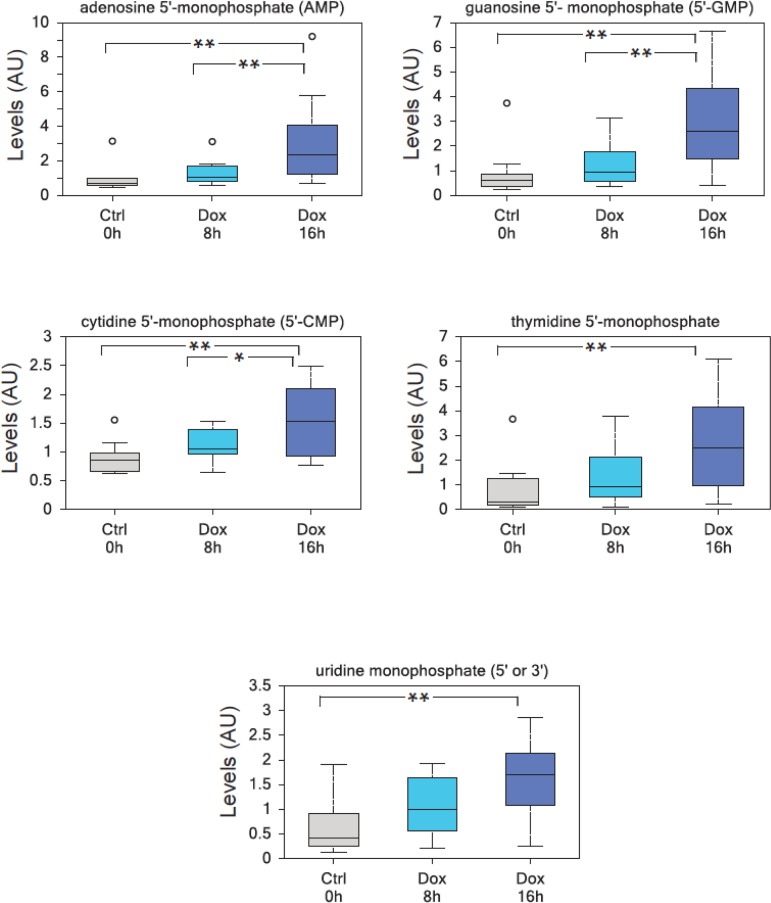
TAp73 overexpression induces nucleotide monophosphates TAp73β overxpression in SaOs-2 Tet-On cell lines results in a highly significant enrichment in all nucleotide monophosphates, consistent with an increased metabolic demand to sustain cell growth. (a) adenosine 5′-monophosphate (AMP), (b) guanosine 5′-monophosphate (5′-GMP), (c) cytidine 5′-monophosphate (5′-CMP), (d) thymidine 5′-monophosphate, (e) uridine monophosphate (5′ or 3′-UMP). Analysis was performed on thirty million cells per samples, 10 samples were analyzed for each time point (n=10). All the samples were extracted using standard metabolic solvent extraction methods and analyzed through GC/MS and LS/MS as previously described [44]. Box indicates upper/lower quartile, bars max/min of distribution. ** p<0.05; * 0.05<p<0.10.

To investigate the effects of TAp73 expression on cellular metabolism, we used human p53/p73 null SaOs-2 osteosarcoma cell line, engineered to overexpress HA-tagged TAp73β isoform when cultured in the presence of the tetracycline analog doxycycline (Dox)[43] and used GC-MS and LC-MS-MS platforms to perform high throughput metabolomics [44]. With this approach, we unveiled an unexpected role for TAp73 in promoting the Warburg effect (manuscript in preparation). TAp73-expressing cells show an increased rate of glycolysis, higher amino acid uptake and increased levels and biosynthesis of acetyl-CoA (manuscript in preparation). Moreover, TAp73 expression increases the activity of several anabolic pathways including polyamine and membrane phospholipid synthesis (manuscript in preparation). In addition, nucleotide biosynthesis was significantly upregulated by TAp73. The biochemical analysis of intracellular nucleotides content is shown in Figures 1 and illustrates a sustained and significant upregulation of both purines and pyrimidines. Thus, our data indicate that TAp73 regulates multiple metabolic pathways that impinge on numerous cellular functions, but which, overall, converge to sustain “biochemical” cell growth and proliferation, in full agreement with the indicated report [35, 45].

### p73 induces cell cycle arrest and cell death

**Figure 2:**
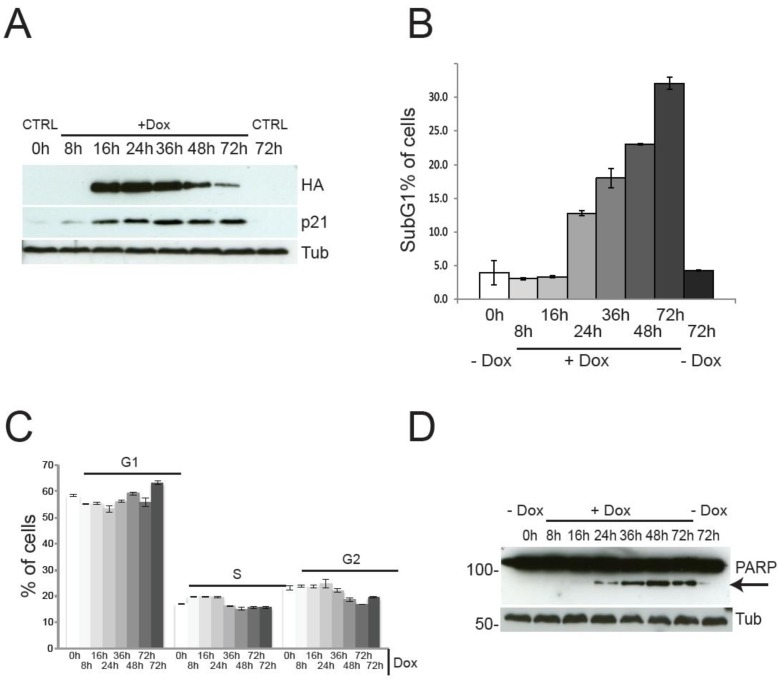
Cell cycle progression and cell death by TAp73β expression TAp73 overexpression in SaOs-2 Tet-On cell lines does not induce proliferation. (**a**) PARP1-cleavage (arrow) induced by TAp73β-expression confirms induction of cell death at 24h after doxycycline (2 μg/ml) addiction. (**b**) Cell death assessed by sub-G1 population after PI staining. Induction of cell death is evident only after 24h of TAp73 induction. Data indicate average of triplicates and standard deviation. Blue arrows indicate the time points used for metabolomics analysis (same cultures as shown here). (**c**) Cell cycle profile after induction of TAp73β determined by PI staining and cytofluorimetric analysis. Controls were left untreated (0h) or treated with vehicle for 72h to account for changes induced by confluence. Data indicate average of triplicates and standard deviation. Blue arrows indicate the time points used for metabolomics analysis (same cultures as shown here). (**d**) TAp73β and p21 expression were assessed by western blotting after treatment with doxycycline (2μg/ml) for the indicated times. TAp73 expression was detected using HA antibody to the N-terminal HA tag. Tubulin was used as loading control. Controls are as in Figure 1.

Although the described findings might be interpreted as suggestive of pro-proliferative function for TAp73, a careful analysis of the cell cycle profile indicates the complete absence of TAp73-induced proliferation. Indeed, Figure 2 shows the cell cycle and the cell death analysis at different time points. Expression of TAp73 β C-terminal isoforms reached plateau after 16h of Dox treatment, without any discernible effect on cell cycle distribution (Figure 2C), except for a mild increased in the G1 phase at 72h post-induction. Notwithstanding, expression of TAp73β was accompanied by a robust upregulation of the cell cycle inhibitor p21, evident already 8h after Dox administration (Figure 2D), strongly arguing against a proliferative role for TAp73. As expected, at later time points, the cells underwent programmed cell death, as previously described for TAp73 [43, 46]. Of note, the timing of the metabolomic analysis (blue arrows) was deliberately chosen before the onset of cell death, to avoid any confusion arising from metabolic changes associated with apoptosis. Overall, these data suggest that, although TAp73 expression stimulates anabolic pathways, it is unlikely to promote cellular proliferation, due to upregulation of the cell cycle inhibitor p21 and induction of apoptosis.

### TAp73 depletion affects nucleotide metabolism in vivo

Since TAp73 exerts a relevant role in the physiology of the nervous system [38, 47-49], we analyzed cerebral cortex and hippocampus isolated from TAp73 wild-type (WT) and knockout (KO) animals [50] to question whether TAp73 regulates nucleotides metabolism in-vivo. Notably, in agreement with the in-vitro experiment, we found that purine metabolism was altered in TAp73KO brains. In particular, inosine and adenosine were significantly higher in the cortex of TAp73KO compared to WT controls. Moreover, allantoin, the final product in purine catabolism, was higher in both TAp73KO cortex and hippocampus, reaching statistical significance in the latter. On the other hand, inosine 5′- monophosphate and adenosine 5′- monophosphate were significantly reduced in the cortex and hippocampus of TAp73 KO mice, respectively. Hence, these data suggest that TAp73 regulates nucleotides metabolism in-vivo, in physiological context.

## DISCUSSION

The identification of extensive metabolic re-arrangements that sustain cancer growth has spurred interest towards a deeper understanding of the underpinning regulatory mechanisms [10, 19, 37, 51-54]. It is widely implied that oncogenes reprogram metabolism to sustain cell growth, whereas tumor suppressors halt malignancy also by mean of metabolic regulation [19, 55, 56]. This network assumes a striking relevance in the case of p53, as impairing p53 family ability to trigger apoptosis [57-64], senescence [65-73] and cell-cycle arrest does not abolish its tumor suppressor efficacy [74-78], which apparently is maintained through regulation of metabolic genes[50, 79-83]. This suggests that metabolism might have greater relevance than previously thought in repressing cellular transformation.

**Figure 3:**
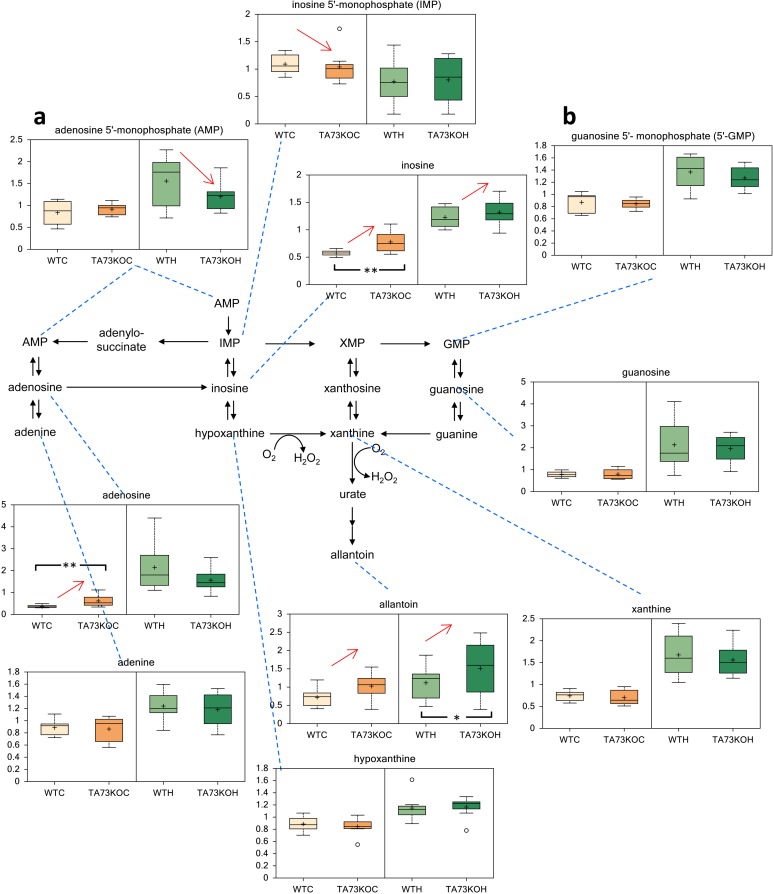
Metabolic Analysis of TAp73 KO mouse Cortex and Hippocampus Nucleotide metabolism in TAp73 knockout (TA73KO) versus wild-type (WT) mouse cerebral cortex (C) and hippocampus (H) (n=8 biological littermate replicates; age 1 day). These are the two areas of the central nervous system that show developmental defects in the knockout mice. (a) adenosine 5′-monophosphate (AMP), (b) guanosine 5′-monophosphate (5′-GMP). Box indicates upper/lower quartile, bars max/min of distribution. * p<0.05.

TAp73 has been known as a tumor suppressor gene able to induce cell cycle arrest and apoptosis [30, 84] similarly to its sibling p53 [40, 85-91]. This view has been recently challenged by the finding that TAp73 promotes cellular proliferation through the expression of the PPP enzyme G6PD and, therefore, diverts glucose metabolism towards PPP and production of NADPH for ROS detoxification and ribose for nucleotide biosynthesis and proliferation [35]. In the attempt to unravel the link between TAp73 and regulation of cellular metabolism, and to understand its association with the established tumor suppressor role of TAp73, we have performed metabolic analysis in-vitro, in cells overexpressing TAp73β and in-vivo in mice depleted of TAp73. We did not observe evident differences in PPP, but we did record increase glycolytic rate in TAp73 expressing Saos-2 cells, together with augmented uptake of amino acids and increased biosynthesis of acetyl-CoA (manuscript in preparation). Moreover we observed a robust increased in intracellular content of nucleotides. Altered metabolism of nucleotides was also identified in in the cortex and hippocampus of TAp73 depleted mice, underlining the physiological relevance of TAp73-mediated control of metabolism. These data are partially in agreement with the findings of Du and colleagues [35]. But the interpretation that TAp73 promotes proliferation [35] would represent a paradigm shift for the p53 family [40, 85, 92] and would hardly reconcile with the ability of TAp73 to regulate expression of the cell cycle inhibitor p21 and to induce apoptosis. Indeed, our data and work from other groups have consistently demonstrated that TAp73 does halt cell cycle and induce cell death in a variety of cells and in response to diverse stimuli, acting as a proper tumor suppressor [24, 29, 43, 85, 92]. Therefore, we question whether the experimental data are sufficiently robust to support this change of dogma.

On the other hand, we have recently demonstrated that TAp73 knockout mice are affected by an aging phenotype accompanied by decreased mitochondrial function, augmented intracellular ROS levels and sensitivity to oxidative stress [31]. These metabolic alterations ultimately converge in promoting accelerated senescence in vitro and aging in-vivo. Therefore, the regulation of cellular metabolism by TAp73 could be interpreted on the light of its anti-senescence and anti-ageing function. This interpretation is reinforced by the finding that cellular senescence suppresses nucleotide metabolism [93, 94]. We could therefore envisage a scenario where TAp73 expression leads to cell cycle arrest or cell death, but promotes a metabolic rewiring that prevents normal cell from undergoing senescence, with possible important implication for neuronal development and neurodegenerative disease, on the light of TAp73 involvement in brain physiology [38, 95].

In summary, our interpretation of the apparent inconsistency, whereby TAp73 promotes “biochemical proliferation” and “cellular cell death”, inhibiting tumor progression, is that TAp73 counteracts cellular senescence by activating an anti-senescence metabolic response.

## MATERIALS AND METHODS

### Cells culture

SaOs-2 Tet-On inducible for TAp73 were cultured at 37 °C in 5% CO_2_ in RPMI 1640 medium (Gibco), supplemented with 10% FCS, 250 mM L-glutamine, penicillin/streptomycin (1 U/ml), and 1 mM pyruvate (all from Life Technologies). TAp73 expression was induced by addition of doxycycline (Sigma) 2μg/ml (stock 2mg/ml in PBS) for the indicated time.

### Western Blots

Proteins were extracted from cell pellets using RIPA buffer (25mM Tris/HCl pH 7.6, 150mM NaCl, 1% NP-40, 1% sodium deoxycholate, 0.1% SDS) supplemented with phosphatase and protease inhibitor cocktails (ROCHE). Quantification of protein extracts was performed using BCA protein assay from PIERCE. 40μg of protein were boiled for 6 minutes at 90°C and then separated using SDS-PAGE, transferred to nitrocellulose membranes using standard transfer techniques and blocked with 5% milk for 2h at room temperature. Primary antibodies were incubated O/N at 4°C in blocking with gentle agitation. We used rabbit HA (Santa Cruz, Y11), rabbit β-tubulin (Santa Cruz, H-135), rabbit p21 (H-164) and Alexis anti-PARP. Horseradish peroxidase (HRP)-conjugated secondary antibodies (BioRad) and ECL chemoluminescence substrate (PIERCE) were used for final detection.

### Metabolic analysis

TAp73 SaOs-2 Tet-On cell lines were cultured in growing medium and treated for 8h and 16h with doxycycline 2 μg/ml to induce TAp73β expression. Control cells were treated with vehicle (PBS) for 16h. Thirty million cells were spun down and pellets were washed once with cold PBS before being frozen in dry ice. All the samples were extracted using standard metabolic solvent extraction methods and analyzed through GC/MS and LS/MS as previously described [44]. After log transformation and imputation with minimum observed values for each group, the comparison of the metabolic compounds of the indicated samples was visualized.

### Cell cycle and survival

For cell cycle analysis 500,000 cells were treated for the indicated time with doxycycline 2μg/ml, collected and fixed with ice cold 70% ethanol. After O/N fixing at −20°C, cells were washed in PBS, resuspended in 50μl of 10μg/ml RNase solution (SIGMA) and incubated for 10 minutes at 37°C. 500μl of staining solution (50μg/ml propidium iodide in PBS) was added to the cells, followed by additional incubation 30 minutes at 37°C. Stained cells were analyzed by flow cytometry and at least 10,000 cells per sample were collected. Data were analyzed using CELLQuest acquisition/analysis software.

### Mice

TAp73 null mice in C57BL6 background were genotyped as previously described [24]. For metabolic analysis cortex and hippocampus were removed from 1 day old mice of both genotype and immediately frozen and stored at −80° C.

The animal experiments were performed under project licenses PPL 40/3442, granted to MA by the UK Home Office. Animal husbandry and experimental design met the standards required by the UKCCCR guidelines.

## Abbreviations

GC: Gas chromatography
MS: Mass Spectrometry
LC: Liquid chromatography
DMED: Dulbecco minimal essential medium
FBS: foetal bovine serum
